# Editorial to “Safety and feasibility of atrial fibrillation ablation after left atrial appendage closure: A single‐center experience of the left atrial appendage closure‐first strategy”

**DOI:** 10.1002/joa3.13097

**Published:** 2024-06-18

**Authors:** Yusuke Kondo, Satoko Ryuzaki, Yoshio Kobayashi

**Affiliations:** ^1^ Department of Cardiovascular Medicine Chiba University Graduate School of Medicine Chiba Japan; ^2^ Department of Advanced Arrhythmia Bioengineering Chiba University Graduate School of Medicine Chiba Japan

**Keywords:** atrial fibrillation, left atrial appendage closure, left atrial appendage occlusion, WATCHMAN

Editorial comment on “Safety and feasibility of atrial fibrillation ablation after left atrial appendage closure: A single‐center experience of the left atrial appendage closure‐first strategy.”^1^

For patients with symptomatic nonvalvular atrial fibrillation (NVAF), physicians usually recommend atrial fibrillation ablation to alleviate symptoms. However, many patients hope for a life without the need for anticoagulation therapy. This expectation can increase the risk of stroke, because stopping anticoagulation therapy against clinical guidelines may lead to postablation asymptomatic NVAF recurrence. Moreover, several issues are associated with anticoagulation therapy, such as difficulties in continuing therapy due to bleeding complications or recurrent thromboembolism despite appropriate therapy. The WATCHMAN device has been available in Japan since 2019. This device was approved by the FDA to reduce the risk of thromboembolism originating from the left atrial appendage (LAA) in patients who have a valid reason to avoid oral anticoagulation therapy. According to the JCS/JHRS guidelines, left atrial appendage closure (LAAC) may be considered a Class IIb indication for patients with NVAF who require thromboembolism prevention and for whom a long‐term alternative to anticoagulation therapy is being considered.^2^ In recent years, favorable outcomes of LAAC have been reported in Japan. However, there is no consensus regarding the optimal postoperative antithrombotic therapy regimen.^3^, ^4^ Additionally, reports on the effectiveness and safety of LAAC following catheter ablation (CA) are scarce.

Chatani et al. found no differences in procedure‐related and cardiovascular adverse events between percutaneous LAAC and percutaneous CA for NVAF.^1^ Furthermore, after both procedures were completed, the LAAC‐first group experienced no device‐related adverse events, such as device‐related thrombus, new peri‐device leakage, progressive increase in peri‐device leakage, or device dislodgement. In contrast, the CA‐first group experienced four device‐related adverse events. Additionally, the primary reason for performing CA after LAAC was heart failure events. The main reason for opting for LAAC first was the presence of patients with a history of LAA thrombosis or LAA sludge who had also experienced major bleeding or were at a high risk of bleeding, as indicated by a HAS‐BLED score of 3 or higher.

While it is ideal to perform both procedures simultaneously, there are limited data comparing the implantation of the WATCHMAN device with anticoagulation therapy. Additionally, the specific disadvantages of simultaneous procedures have not been thoroughly investigated.

The OPTION study will assess whether LAAC with the WATCHMAN FLX device is a viable alternative to oral anticoagulation therapy following CA for NVAF.^5^ This is a multinational, multicenter, prospective, randomized clinical trial. Patients with a CHA2DS2‐VASc score ≥2 in men or ≥3 in women who underwent CA for NVAF between 90 and 180 days before randomization (sequential) or are scheduled to have CA within 10 days of randomization (concomitant) will be randomized in a 1:1 ratio to receive either the WATCHMAN FLX or a control treatment. Control patients will initiate or continue market‐approved oral anticoagulation therapy for the duration of the trial. In total, 1600 patients were randomized from 130 investigational sites worldwide. Follow‐up for both the device and control patients will be conducted at 3, 12, 24, and 36 months. The primary effectiveness noninferiority endpoint is the occurrence of stroke (ischemic or hemorrhagic), all‐cause mortality, or systemic embolism at 36 months. The primary safety superiority endpoint is nonprocedural bleeding for up to 36 months (including International Society on Thrombosis and Hemostasis [ISTH] major bleeding or clinically relevant nonmajor bleeding). The secondary noninferiority endpoint is ISTH major bleeding within 36 months (including procedural bleeding). The primary effectiveness noninferiority endpoint is stroke (ischemic or hemorrhagic), all‐cause death, or systemic embolism at 36 months. The primary safety superiority endpoint is nonprocedural bleeding over 36 months (ISTH major bleeding or clinically relevant nonmajor bleeding). The secondary noninferiority endpoint is ISTH major bleeding over 36 months (including procedural bleeding).

Performing LAAC and CA simultaneously using intracardiac echocardiography (ICE) under local anesthesia offers significant advantages, as it makes LAAC feasible even in cases where transesophageal echocardiography cannot be performed or when general anesthesia is challenging. However, in this concurrent procedure, various issues must be addressed, including the selection of the appropriate ablation energy and the formulation of postoperative antithrombotic therapy regimens. Moreover, regarding LAAC guided by ICE, the procedure is complex; thus, it is necessary to establish the safety of the technique using ICE before conducting an OPTION trial in Japan. Nevertheless, the establishment of optimal management for stroke prevention in patients with NVAF is anticipated.

## FUNDING INFORMATION

None.

## CONFLICT OF INTEREST STATEMENT

Dr. Kondo received lecture fees from Daiichi‐Sankyo, Bayer, Abbott Medical Japan, Biotronik Japan, and Boston Scientific, Japan. Dr. Ryuzaki belongs to an endowed department supported by Medtronic, Japan. Dr. Kobayashi received lecture fees from Abbott Medical Japan, Bayer Japan, Bristol‐Myers Squibb, Boehringer Ingelheim, and Daiichi‐Sankyo, and scholarship funds from Takeda Pharmaceutical, Abbott Medical Japan, Terumo, Otsuka Pharmaceutical, Boehringer Ingelheim, Astellas, Daiichi‐Sankyo, Win International, Japan Lifeline, and Nipro.

## CLINICAL TRIAL REGISTRATION

N/A.

## ETHICS STATEMENT

N/A.

## PATIENT CONSENT STATEMENT

N/A.

## Data Availability

The deidentified participant data will not be shared.
